# Evaluation of the patient acceptable symptom state following hip arthroscopy using the 12 item international hip outcome tool

**DOI:** 10.1186/s12891-019-3026-x

**Published:** 2020-01-03

**Authors:** Patrick G. Robinson, Julian F. Maempel, Conor S. Rankin, Paul Gaston, David F. Hamilton

**Affiliations:** Trauma and Orthopaedic Department, University of Edinburgh, 51 Little France Crescent, Edinburgh, EH16 4SA Scotland

**Keywords:** Femoracetabular, Impingement, Hip, Pyschometric, iHOT-12

## Abstract

**Background:**

The International Hip Outcome Tool 12 (iHOT-12) is a shorter version of the iHOT-33 which measures health related quality of life following treatment of hip disorders in young, active patients. The purpose of this study was identify a PASS threshold for a UK population undergoing hip arthroscopy for intra-articular hip pathology.

**Methods:**

Data was identified retrospectively from a prospective database of patients undergoing hip arthroscopy under the care of a single surgeon within the date range January 2013 to March 2017. All patients with a diagnosis of femoroacetabular impingment (FAI) undergoing arthroscopic treatment were included. iHOT-12, EuroQol 5D-5 L (EQ-5D-5 L) and a satisfaction questionnaire were available pre and post-operatively. PASS was calculated using an anchor-based approach and receiver operator characteristic (ROC) analysis.

**Results:**

171 patients underwent hip arthroscopy in the study period. Linked longitudinal follow-up data was available for 122 patients (71.3%) at a median of 24.3 months (740 days, interquartile range 576–1047). The PASS threshold for the iHOT-12 was 59.5 (sensitivity 81.1%, specificity 83.9%; area under the curve (AUC) 0.92, 95% CI 0.87–0.97). 64% of patients achieved this score. The median postoperative iHOT-12 score was 72.5 (IQR 44) and the mean change in score was 35 (SD 25, *p* < 0.001). The EQ-5D Index improved by 0.18 (SD 0.25, *p* < 0.001) and there was a mean change of 7.67 (SD 24.82) on the EQ-5D VAS (*p* = 0.001).

**Conclusions:**

We report a PASS threshold of the iHOT-12 following hip arthroscopy for FAI as a measurable benchmark for clinicians using this outcome measure.

## Background

Hip arthroscopy has been shown to be an effective treatment modality for the surgical management of femoroacetabular impingement (FAI) and labral injuries [1, 2]. Consequently, there has been a significant increase in the volume of arthroscopic hip surgery performed in recent years and specific patient reported outcome measures (PROMs) have been developed to measure the success of this intervention [3–7]. This has been particularly important for high functioning, young, active patients, as existing outcome measures were typically designed for older or less active cohorts. As such, they frequently suffer from ceiling effects that impair the assessment of meaningful change in highly performing groups – such as those undergoing hip arthroscopy [8, 9].

The aim of the original international hip outcome tool 33 (iHOT-33) was to produce an outcome tool that measures health-related quality of life and took into consideration young patients’ views on what they felt was important following surgery [3]. Two recent systematic reviews have reported the iHOT to be favourable compared to other hip-specific outcome measures with regards to pyschometric properties [10, 11]. However, the iHOT-33 was primarily designed for research purposes and was felt to be too long and burdensome for day to day clinical practice and therefore the iHOT-12 was developed [4]. The iHOT-12 uses 12 questions from the original 33 and accounts for 96–99% of the total variation of the full score, based on regression analysis, and covers all four domains of the iHOT-33 [4]. It is now included as part of the minimal dataset in both the British and Swedish non-arthroplasty hip registries [12, 13] and has been validated in many languages [14–16]. Despite this, there are still uncertainties as to what particular postoperative scores actually mean to the patient.

The responsiveness of a scoring tool is judged by its’ ability to distinguish change when a change in status has indeed occurred. The patient acceptable symptomatic state (PASS) score has been defined as a postoperative outcome score threshold, above which a patient is deemed to have had a satisfactory outcome [17] and it has previously been used as a responsiveness tool for total hip and knee replacement [18–20]. The PASS score is typically reported as an absolute value, as opposed to a change in value. The benefit of this is that a predetermined PASS score may be compared with PROMs at any given time point. Two recent studies have published contrasting PASS thresholds for postoperative iHOT-12 scores [21, 22] which may attributed to differences in the methodology of the psychometric analysis.

The purpose of this study was to evaluate a PASS score threshold for the iHOT-12 for patients undergoing hip arthroscopy surgery for femoroacetabular impingement.

## Methods

Patients for this study were identified retrospectively from a prospective database of patients undergoing hip arthroscopy by a single surgeon within the date range January 2013 to March 2017. All patients diagnosed with FAI undergoing primary hip arthroscopy procedures for labral tears during this time frame were included. Included patients had been diagnosed by the treating surgeon with FAI (using clinical history, examination, plain radiographs and magnetic resonance arthrogram if appropriate) and had failed a trial of non-operative treatment including analgesia and physiotherapy. Joint injections were used to confirm the origin of symptoms in cases of doubt. The hip joint capsule was not repaired. Patients completed preoperative EQ-5D-5 L and iHOT-12 questionnaires 2 weeks prior to surgery at the pre-assessment clinic and at a minimum of 1 year post operatively. Satisfaction data was collected at a minimum of 1 year postoperatively.

### Outcome measures

The EQ-5D-5 L consists of an EQ-5D index, comprising five domains (mobility, self-care, usual activities, pain/discomfort, and anxiety/depression), with scores ranging from − 1 to + 1, and an EQ-5D visual analog scale (VAS), on which patients self-rate their health state between 0 and 100. The iHOT-12 consists of four domains; symptoms and functional limitations, sport and recreational activities, job-related concerns, and social, emotional, and lifestyle concerns. Each item in the iHOT-12 was scored using a visual analog scale from 0 to 100, with a score of 100 being the best function and least amount of symptoms, and the overall mean equates to the final iHOT score.

An anchor-based approach was used to establish the PASS. The anchor question used was modified from that designed by Tubach et al. [23]. We asked patients “How satisfied are you following your surgery?” The response to the question was graded using a 5-point Likert scale: very satisfied, satisfied, neither satisfied or dissatisfied, dissatisfied or very dissatisfied. We dichotomized responses for analysis, accepting ‘satisfied’ and ‘very satisfied’ responses as positive. We then used two validation questions “would you have this operation again if it was required on another joint?” and “would you recommend similar treatment to your friends or family?” The latter question also forms the basis of the family and friends test (FFT) in the English National Health Service [24]. The purpose of the validation questions was to further qualify the responses to the satisfaction question and to determine whether the addition of these variables changed the threshold score for the PASS. Both questions were answered on a 5-point Likert scale: extremely likely, likely, neither likely nor unlikely, unlikely or extremely unlikely. We deem a positive response to be ‘extremely likely’ or ‘likely’.

Receiver operating characteristic (ROC) curves to calculate iHOT-12 PASS thresholds were performed for a composite of a positive response to the satisfaction question and a positive response to the question regarding willingness to undergo the same operation if required. We then performed a further ROC analysis, adding the cohort of patients who also responded positively to the family/friend recommendation question, to the previous composite score. We also performed secondary exploratory analysis to calculate a PASS threshold for change in iHOT-12 score using ROC curves.

### Statistical analysis

Statistical analysis was undertaken using Statistical Package for Social Sciences (SPSS) software (IBM, Inc., Armonk, New York, United States) v24. Normality was assessed using Kolmogorov-Smirnov testing. Continuous, normally distributed data was reported as mean with standard deviation and was compared using 2-tailed student t-tests. Non-parametric data was reported as median with interquartile range and compared using Mann Whitney U-tests. Cross-tabulated data for dichotomous variables were analyzed using chi squared tests. A *p*-value of <0.05 was considered statistically significant.

ROC curve analysis was used to identify thresholds for the iHOT-12 score (absolute and change in score) that predicted the outcome variables. The area under a ROC curve ranges from 0.5, indicating a test with no accuracy in distinguishing the outcome variable (e.g. satisfaction), to 1.0 where the test would be perfectly accurate at identifying the outcome variable in all patients. The threshold is equivalent to the point (iHOT-12 score) at which sensitivity and specificity are maximal in predicting the outcome variable. A ROC curve with an area under the curve of >0.7 is considered to demonstrate a test with acceptable discriminatory power and > 0.8 is considered excellent [25]. For the purposes of dichotomous statistical analysis pertaining to satisfaction, patients stating they were ‘very satisfied’ or ‘satisfied’ were considered satisfied while all other responses were considered unsatisfied. For the purposes of dichotomous statistical analysis pertaining to willingness to undergo the same procedure again and recommendation to others, the responses ‘extremely likely’ and ‘likely’ were considered likely while all other responses were not.

## Results

171 patients underwent hip arthroscopy at our institution in the period under review, 122 patients (71.3%) completed post-operative PROMs scores at a median follow up of 740 days (Interquartile range 576–1047) (24.3 months). The specific surgical procedures performed are detailed in Table 1. There were no differences in age, gender, pre-op iHOT-12 score or pre-op EQ-5D VAS between those who did and did not respond to follow-up questionnaires (Table 2). The median postoperative iHOT-12 score was 72.5 (IQR 44). The median postoperative EQ-5D index score was 0.767 (IQR 0.307) and the postoperative VAS score was 80 (IQR 30).

**Table 1:** Summary of procedures performed

	n
Acetabular procedures	
Labral repairs	
With or without rim recession	97
With microfracture with or without rim recession	16
With psoas bursa excision	1
With removal of os acetabuli	1
Labral resection	
With or without rim recession	20
With microfracture and rim recession	11
With removal of loose body	1
Acetabular procedure not recorded	1
Femoral procedure	
Cam removal	
Isolated cam removal	102
With osteophyte removal	8
With microfracture	2
With decompression of impingement cyst	1
With psoas release	1
With drilling of femoral head for avascular necrosis	1
Loose body removal	1
No femoral procedure performed	22
Femoral procedure not recorded	1

**Table 2:** Demographics and preoperative scores of responders and non-responders

	Responders	Non-Responders	Comparison responders vs non-responders	Whole group
Age	28 yrs. (IQR 14)	31 yrs. (IQR 9)	0.383	29 (IQR 13)
Gender	76 female: 46 male	25 female: 24 male	0.175	101 female: 70 male
BMI	24.69 kg/m^2^ (SD 3.76)	24.32 kg/m^2^ (SD 3.24)	0.610	24.58 kg/m^2^ (SD 3.61)
Pre-op iHOT-12	31.52 (SD 15.78)	31.55 (SD 15.5)	0.991	31.53 (SD 15.65)
Pre-op EQ-5D Index	0.647 (IQR 0.287)	0.564 (IQR 0.303)	0.299	0.635 (IQR 0.298)
Pre-op EQ-5D VAS	70 (IQR 30)	75 (IQR 33)	0.34	70 (IQR 30)

### Change Between Pre-op and Post-op

There was a mean change of 35.02 (SD 25.08) points in the iHOT-12 score between pre- and post-operative review (*p* = <0.001). Using the method of one half of the standard deviation of the difference in pre and post-operative outcome score to quantify the minimally clinical important difference (MCID), we found this to be 12.54. The EQ-5D Index improved by a mean of 0.18 (SD 0.25) at the time of post-operative review (*p* < 0.001) and there was a mean change of 7.67 (SD 24.82) on the EQ-5D VAS between pre- and post-operative review (*p* = 0.001).

### Satisfaction and iHOT-12

One hundred twenty-one patients answered the satisfaction question. Of these, 43 stated they were very satisfied, 47 satisfied, 17 neither satisfied nor dissatisfied, 12 dissatisfied and 2 very dissatisfied. As such 90 (74.4%) respondents were coded as satisfied for analysis. Satisfied patients had greater mean improvement in iHOT-12, EQ-5D index and EQ-5D VAS than unsatisfied patients (Table 3).

**Table 3:** Change in pre and postoperative iHOT-12 scores, EQ-5D Index and EQ-5D VAS for patients who were satisfied or unsatisfied

	Satisfied patients	Unsatisfied patients	*P*-value
Change in iHOT-12	+ 43.20 (SD 20.73)	+ 12.85 (SD 21.96)	<0.001
Change in EQ-5D Index	+ 0.23 (SD 0.210)	+ 0.06 (SD 0.306)	0.006
Change in EQ-5D VAS	+ 13.77 (SD 20.42)	−7.77 (SD 27.07)	<0.001

There was no significant difference in age or gender distribution between those who were satisfied and those who were not satisfied, however there was a difference in pre-operative iHOT-12 score (Table 4).

**Table 4:** Demographics and preoperative iHOT-12 scores for patients who were satisfied or unsatisfied

	Satisfied patients	Unsatisfied patients	p-value
Age	29 yrs. (IQR 14)	27 yrs. (IQR 17)	0.845
Gender Distribution	52 female: 38 male	24 female: 7 male	0.051
Pre-op iHOT-12	34.41 (SD 15.70)	23.75 (SD 13.19)	0.001

### Pass for iHOT-12

**Fig. 1:**
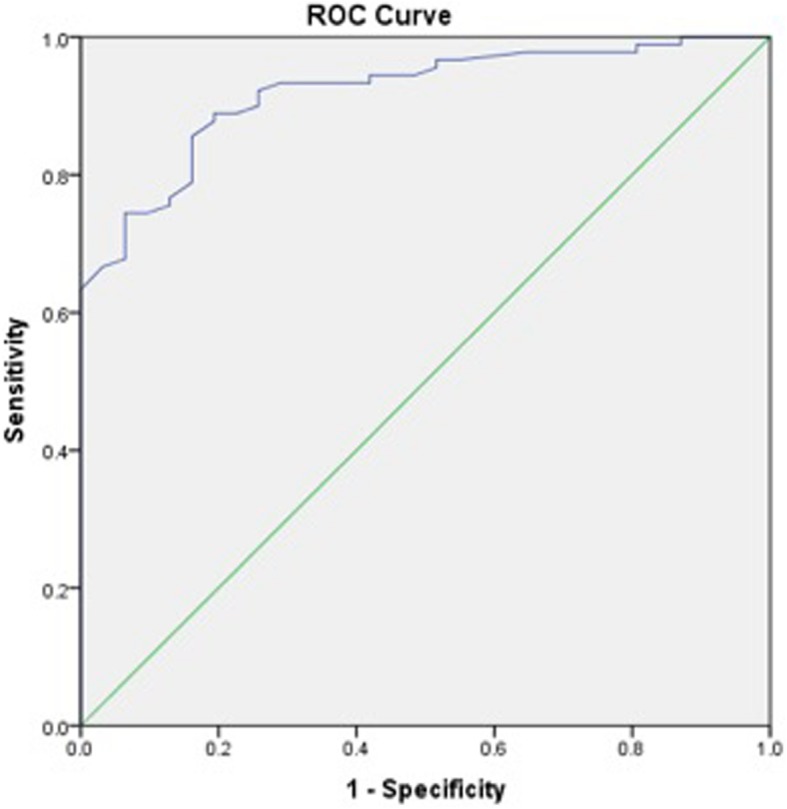
Receiver Operator Characteristic (ROC) curve for the threshold score of the iHOT-12 to determine satisfaction

**Fig. 2:**
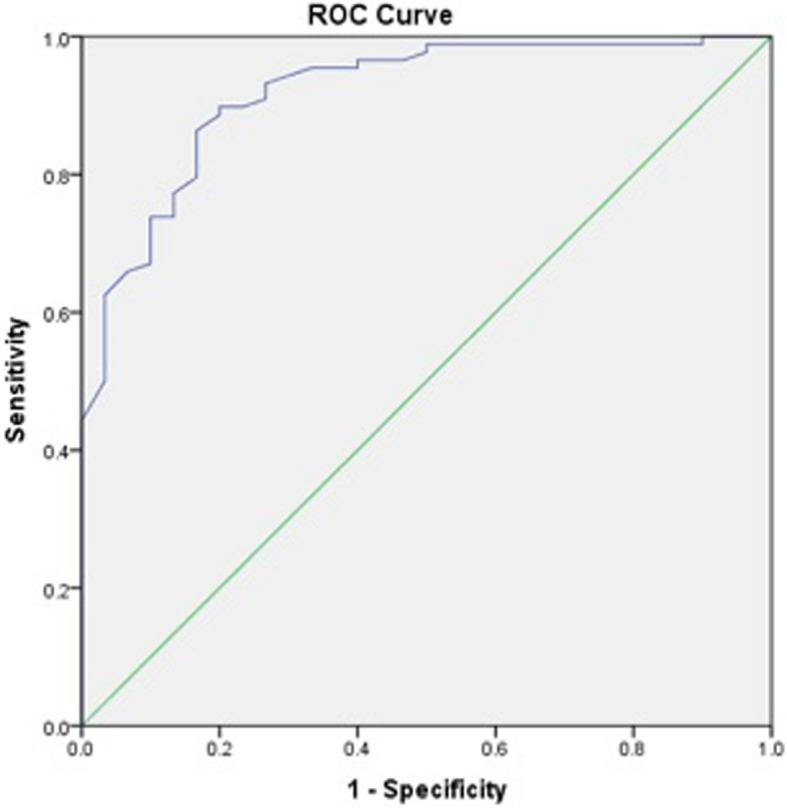
Receiver Operator Characteristic (ROC) curve for the threshold score of the iHOT-12 to determine satisfaction and willingness to have the same operation again, if required, on another joint

**Fig. 3:**
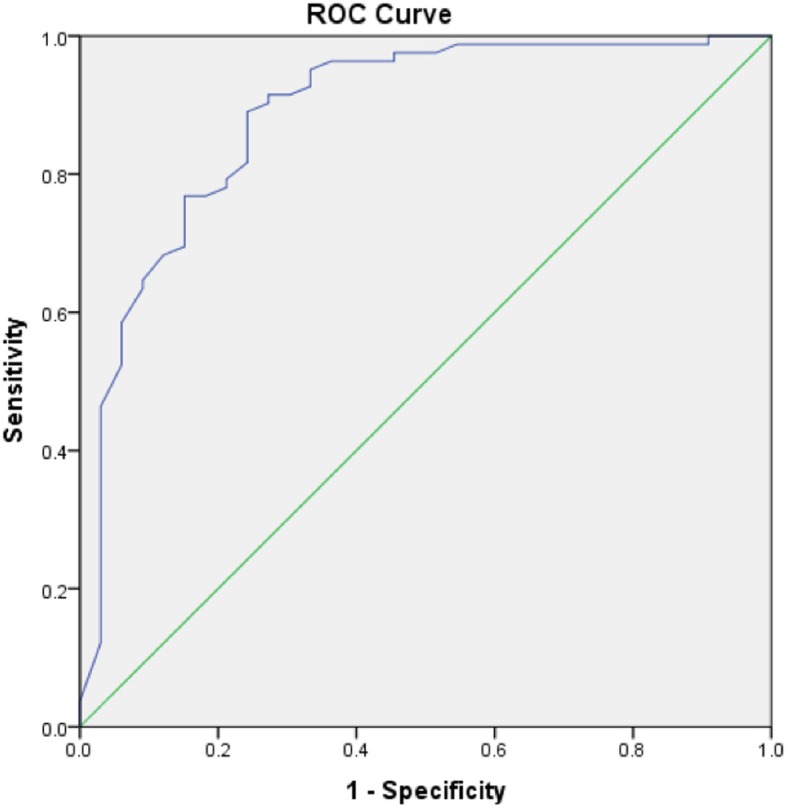
Receiver Operator Characteristic (ROC) curve for the threshold score of the iHOT-12 to determine satisfaction and willingness to have the same operation again, if required, on another joint and likelihood of referring your friend or family member for similar treatment

The calculated PASS value for the absolute iHOT-12 score at a median 24.3 months (740 days IQR, 576–1047) post-operatively was 59.5 (sensitivity 81.1%, specificity 83.9%). The area under the curve (AUC) was 0.92 (95% CI 0.87–0.97) (Fig. 1). There were a total of 78 (64%) patients who achieved the PASS score threshold. Exploratory analysis of more complex threshold criteria, including willingness to undergo the procedure again did not affect the PASS value (iHOT score 59.5 [sensitivity 81.8%, specificity 83.3%]. The AUC was 0.92 (95% CI 0.86–0.97) (Fig. 2). Whereas, adding a positive response from the family/friend recommendation question to the previous composite score did increase the required iHOT-12 score. The PASS threshold increased to 64.0 (sensitivity 78%, specificity 78.8%). The AUC was 0.89 (95% CI 0.81–0.96) (Fig. 3).

**Fig. 4:**
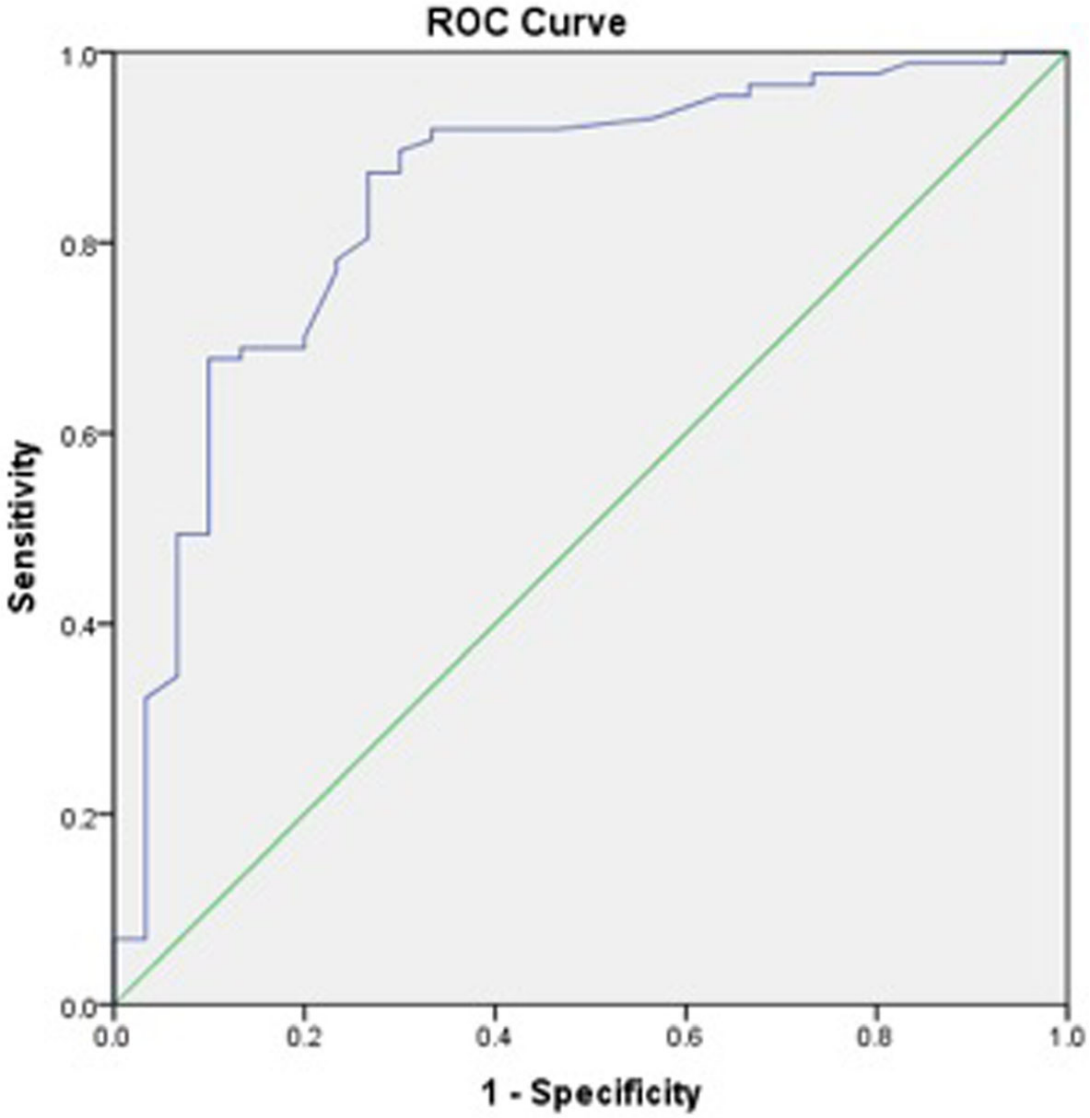
Receiver Operator Characteristic (ROC) curve for the threshold change in score of the iHOT-12 to determine satisfaction

**Fig. 5:**
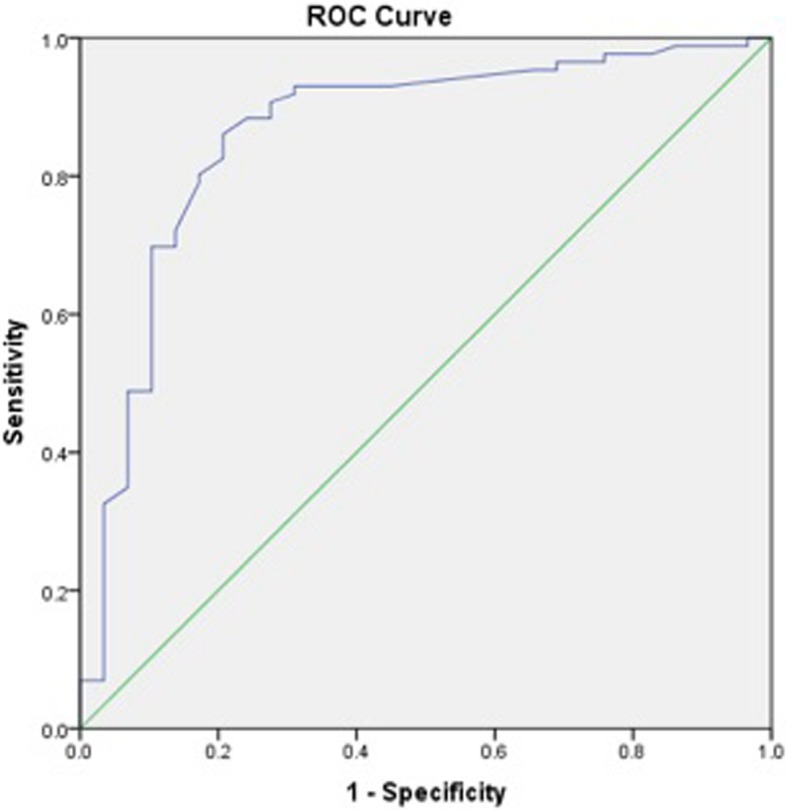
Receiver Operator Characteristic (ROC) curve for the threshold change in score of the iHOT-12 to determine satisfaction and willingness to have the same operation again, if required, on another joint

**Fig. 6:**
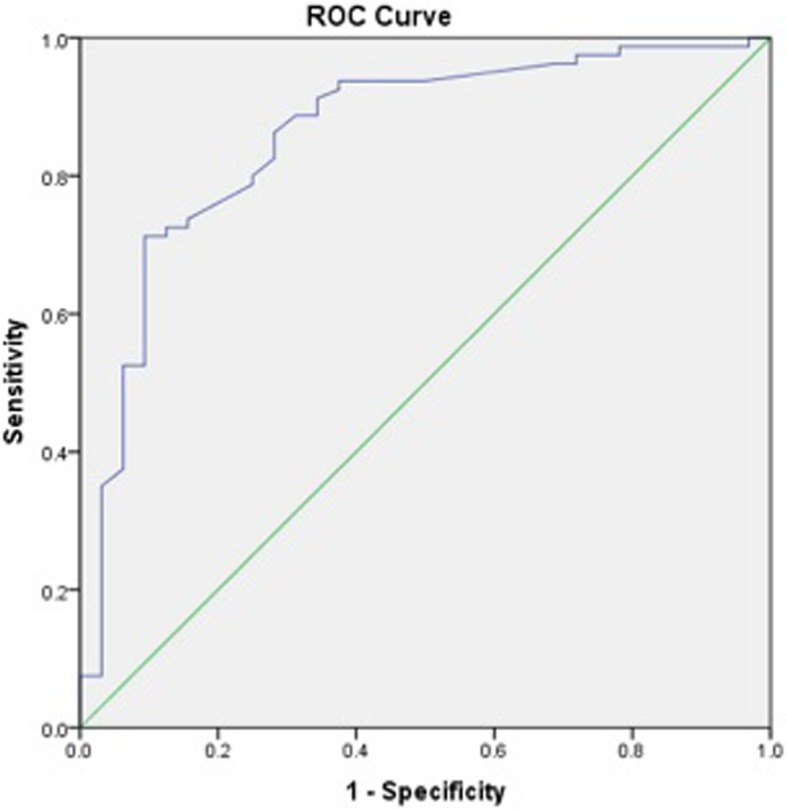
Receiver Operator Characteristic (ROC) curve for the threshold change in score of the iHOT-12 to determine satisfaction and willingness to have the same operation again, if required, on another joint and likelihood of referring your friend or family member for similar treatment

A variant of the PASS value was also calculated for the change between pre and postoperative iHOT-12 scores. This was 31.5 points (sensitivity 70.1%, specificity 80%). The AUC was 0.85 (95% CI 0.76–0.93) (Fig. 4). The PASS score for satisfaction and a positive response to the likelihood to undergo similar surgery again on another joint was + 28.5 points (Sensitivity = 80.2%, Specificity = 82.8%). The AUC was 0.862 (95% CI = 0.776–0.948) (Fig. 5). Finally, the PASS value for the composite score of satisfaction and the likelihood to under the same operation again, if required, on another joint as well as a positive response to the family/friend recommendation questions was 31.5 (sensitivity 73.8% specificity 84.4%). The AUC was 0.86 (95% CI 0.78–0.94) (Fig. 6).

## Discussion

The most significant finding from this study was the identification of the PASS threshold for the iHOT-12 (59.5) with associated sensitivity and specificity of 81.1 and 83.9% respectively and an AUC of 0.92. Secondary exploratory analysis with additional variables exploring willingness to undergo similar surgery in the future and willingness to recommend the procedure to family and friends were used to validate these findings.

A recent study by Kivlan et al. reported the iHOT-12 PASS threshold to be 75.2. However, only 53% of their patients achieved this score. The authors defined satisfaction as those who met the already established PASS scores of the modified harris hip score (mHHS) and hip outcome score – activities of daily living (HOS-ADL) without the use of an anchor question. The authors believed holding their study to the standards of previously calculated PASS scores for HOS-ADL and mHHS would likely cause their calculation of the iHOT-12 PASS score to be higher. This may explain why we found the proportion of patients in our study who achieved the PASS score to be higher. Differences in the PASS thresholds demonstrate the variability of results when different psychometric methodologies are used. Variations in scores using different methodologies (i.e. anchor based vs distributional) have previously been highlighted in the orthopaedic spinal literature [26–28].

A further study has also calculated the PASS threshold for the iHOT-12 and found it to be 63 [21]. However, in their study the authors used the external anchor question “Taking into account all the activities you have during your daily life, your level of pain, and also your functional impairment, do you consider that your current state is satisfactory?” This score is more in keeping with the score reported in this study however, the slight difference may be explained by the subtle differences in the phrasing of the satisfaction question. This theory has been shown to be valid in the arthroplasty literature when calculating the minimally clinical important difference [29]. The difference may also be explained by differences in cultural perceptions and expectations. We report the results of a UK population and the thresholds reported in this paper may be different for other nationalities.

The calculated PASS threshold in this study did not change when adding in the validation question of willingness to undergo further surgery if required. However, with the addition of “would you recommend similar treatment to your friends or family?” this increased the PASS threshold to 64. It has previously been recommended to utilize multiple anchor questions in psychometric analysis to improve reliability and validate the threshold score being assessed [30]. Discrepancies in satisfaction and the family/friend recommendation question has been reported in a previous study related to hip arthroscopy [31]. The differentiating power of this additional question suggests that patients may be more circumspect as to outcomes when suggesting healthcare interventions to a loved one, and as such this may be a pertinent methodology of evaluating surgical success.

We used distributional methodology to calculate the MCID for the iHOT-12 and found this to be 13 points. This is very similar to the estimates of other studies that have reported the MCID for the iHOT-12 score [21, 22] and although this was not the primary analysis of our study it lends credibility to the representativeness of our underlying dataset.

Patients in this study reported significant changes in their quality of life following surgery, which was demonstrated by increases in their EQ-5D indices and VAS scores. The health-related quality of life scores in this paper rest in between that of two recent randomized controlled trials (RCT) comparing hip arthroscopy to physiotherapy [1, 2]. Patients in our study who reported being satisfied had significantly greater changes in postoperative iHOT-12, EQ-5D and VAS scores compared to those who were unsatisfied. These results confirm that a patient’s perception of their improvement in hip function and health related quality of life is directly related to their satisfaction after hip arthroscopy for FAI. Similar findings have been reported following other orthopaedic procedures [32]. Satisfied patients had a significantly higher preoperative iHOT-12 score of a magnitude consistent with the MCID score, confirming a finding previously reported for the HOS and mHHS [33].

This study must be interpreted in light of its limitations. No consensus has been reached with regards to the gold standard of external anchors when calculating the PASS [23] and this is reflected in the variation in anchor questions which have been used in the medical literature. However, our reported PASS score was similar to another study in this field as previously mentioned. Some loss to follow-up is inevitable in studies of this nature, however, when preoperative data was analyzed for responders and non-responders, no significant differences were noted with respect to age, gender, BMI, preoperative iHOT-12 score, or preoperative EQ. 5D index and VAS scores. This suggests that patients completing follow-up were representative of the overall population undergoing surgery. Finally, this is an experienced, single surgeon series, and although this has kept surgical variables to a minimum, it may not be generalisable to the wider surgical community.

## Conclusion

We report a PASS score of 59.5 points for the iHOT-12 score following hip arthroscopy for FAI in a UK population. Secondary exploratory analysis with additional variables exploring willingness to undergo similar surgery in the future and willingness to recommend the procedure to family and friends were used to validate these findings.

## Data Availability

The datasets used and/or analysed during the current study are available from the corresponding author on reasonable request.

## Abbreviations

AUC: Area under the curve
BMI: Body mass index
CI: Confidence interval
EQ 5D-5 L: EuroQol 5D-5 L
FFT: Family friends test
HOS-ADL: Hip outcome score activities of daily living
iHOT: International hip outcome tool
MCID: Minimally clinical important differenc
mHHS: Modified harris hip score
PASS: Patient acceptable symptomatic state
PROMs: Patient reported outcome measures
RCT: Randomized controlled trial
ROC: Receiver operator curve

## References

[1] Griffin DR, Dickenson EJ, Wall PDH (2018). Hip arthroscopy versus best conservative care for the treatment of femoroacetabular impingement syndrome (UK FASHIoN): a multicentre randomised controlled trial. Lancet.

[2] Palmer AJR, Ayyar Gupta V, Fernquest S (2019). Arthroscopic hip surgery compared with physiotherapy and activity modification for the treatment of symptomatic femoroacetabular impingement: multicentre randomised controlled trial. BMJ.

[3] Mohtadi NG, Griffin DR, Pedersen ME (2012). The Development and validation of a self-administered quality-of-life outcome measure for young, active patients with symptomatic hip disease: the International Hip Outcome Tool (iHOT-33). Arthroscopy.

[4] Griffin DR, Parsons N, Mohtadi NG (2012). A short version of the International Hip Outcome Tool (iHOT-12) for use in routine clinical practice. Arthroscopy.

[5] Martin RL, Kelly BT, Philippon MJ (2006). Evidence of validity for the hip outcome score. Arthroscopy.

[6] Christensen CP, Althausen PL, Mittleman MA (2003). The nonarthritic hip score: reliable and validated. Clin Orthop Relat Res.

[7] Thorborg K, Holmich P, Christensen R (2011). The Copenhagen Hip and Groin Outcome Score (HAGOS): development and validation according to the COSMIN checklist. Br J Sports Med.

[8] Garbuz DS, Xu M, Sayre EC (2006). Patients’ outcome after total hip arthroplasty: a comparison between the Western Ontario and McMaster Universities index and the Oxford 12-item hip score. J Arthroplast.

[9] Marx RG, Jones EC, Atwan NC (2005). Measuring improvement following total hip and knee arthroplasty using patient-based measures of outcome. J Bone Joint Surg Am.

[10] Kemp JL, Collins NJ, Roos EM (2013). Psychometric properties of patient-reported outcome measures for hip arthroscopic surgery. Am J Sports Med.

[11] Thorborg K, Tijssen M, Habets B (2015). Patient-Reported Outcome (PRO) questionnaires for young to middle-aged adults with hip and groin disability: a systematic review of the clinimetric evidence. Br J Sports Med.

[12] Wilson MN, D. (2018). The non-arthroplasty hip registry: 2018 annual report.

[13] Sansone M, Ahlden M, Jonasson P (2014). A Swedish hip arthroscopy registry: demographics and development. Knee Surg Sports Traumatol Arthrosc.

[14] Watanabe Nobuyuki, Murakami Satona, Uchida Soshi, Tateishi Satoshi, Ohara Hidetsugu, Yamamoto Yasuhiro, Kojima Taiki (2019). Exploring the validation of a Japanese version of the International Hip Outcome Tool 12: Reliability, validity, and responsiveness. Journal of Orthopaedic Science.

[15] Baumann F, Popp D, Muller K (2016). Validation of a German version of the International Hip Outcome Tool 12 (iHOT12) according to the COSMIN checklist. Health Qual Life Outcomes.

[16] Stevens M, van den Akker-Scheek I, ten Have B (2015). Validity and Reliability of the Dutch Version of the International Hip Outcome Tool (iHOT-12NL) in Patients With Disorders of the Hip. J Orthop Sports Phys Ther.

[17] Kvien TK, Heiberg T, Hagen KB (2007). Minimal clinically important improvement/difference (MCII/MCID) and patient acceptable symptom state (PASS): what do these concepts mean?. Ann Rheum Dis.

[18] Keurentjes JC, Van Tol FR, Fiocco M (2014). Patient acceptable symptom states after totalhip or knee replacement at mid-term follow-up: Thresholds of the Oxford hip and knee scores. Bone Joint Res.

[19] Dawson J, Fitzpatrick R, Murray D (1998). Questionnaire on the perceptions of patients about total knee replacement. J Bone Joint Surg (Br).

[20] Dawson J, Fitzpatrick R, Carr A (1996). Questionnaire on the perceptions of patients about total hip replacement. J Bone Joint Surg (Br).

[21] Nwachukwu BU, Chang B, Beck EC (2018). How should we define clinically significant outcome improvement on the iHOT-12?. HSS J.

[22] Kivlan BR, Martin RL, Christoforetti JJ (2019). The Patient Acceptable Symptomatic State of the 12-Item International Hip Outcome Tool at 1-Year Follow-Up of Hip-Preservation Surgery. Arthroscopy.

[23] Tubach F, Ravaud P, Beaton D (2007). Minimal clinically important improvement and patient acceptable symptom state for subjective outcome measures in rheumatic disorders. J Rheumatol.

[24] England. N. The friends and family test. London, UK: NHS England 2014 [Available from: https://www.england.nhs.uk/fft/.

[25] Pepe M (2006). The statistical evaluation of medical tests for classification and prediction: Oxford statistical science series.

[26] Gum JL, Glassman SD, Carreon LY (2013). Clinically important deterioration in patients undergoing lumbar spine surgery: a choice of evaluation methods using the Oswestry Disability Index, 36-Item Short Form Health Survey, and pain scales: clinical article. J Neurosurg Spine.

[27] Parker SL, Godil SS, Shau DN (2013). Assessment of the minimum clinically important difference in pain, disability, and quality of life after anterior cervical discectomy and fusion: clinical article. J Neurosurg Spine.

[28] de Vet HC, Ostelo RW, Terwee CB (2007). Minimally important change determined by a visual method integrating an anchor-based and a distribution-based approach. Qual Life Res Int J Qual Life Asp Treat Care Rehab.

[29] Clement ND, MacDonald D, Simpson AHRW (2014). The minimal clinically important difference in the Oxford knee score and short form 12 score after total knee arthroplasty. Knee Surg Sports Traumatol Arthrosc.

[30] Revicki D, Hays RD, Cella D (2008). Recommended methods for determining responsiveness and minimally important differences for patient-reported outcomes. J Clin Epidemiol.

[31] Maempel JF, Ting JZ, Gaston P (2018). Assessing the Outcome of Hip Arthroscopy for Labral Tears in Femoroacetabular Impingement Using the Minimum Dataset of the British Non-arthroplasty Hip Register: A Single-Surgeon Experience. Arthroscopy.

[32] Judge A, Arden NK, Kiran A (2012). Interpretation of patient-reported outcomes for hip and knee replacement surgery: identification of thresholds associated with satisfaction with surgery. J Bone Joint Surg (Br).

[33] Chahal J, Van Thiel GS, Mather RC (2015). The Patient Acceptable Symptomatic State for the Modified Harris Hip Score and Hip Outcome Score Among Patients Undergoing Surgical Treatment for Femoroacetabular Impingement. Am J Sports Med.

